# The Aryl Hydrocarbon Receptor in Chronic Kidney Disease: Friend or Foe?

**DOI:** 10.3389/fcell.2020.589752

**Published:** 2020-12-07

**Authors:** Yenan Mo, Zhaoyu Lu, Lixin Wang, Chunlan Ji, Chuan Zou, Xusheng Liu

**Affiliations:** ^1^The Second Clinical College of Guangzhou University of Chinese Medicine, Guangzhou, China; ^2^Department of Nephrology, The Second Affiliated Hospital of Guangzhou University of Chinese Medicine, Guangzhou, China

**Keywords:** aryl hydrocarbon receptor, chronic kidney disease, tryptophan metabolism, uremic toxin, CKD complications, intestine homeostasis

## Abstract

The aryl hydrocarbon receptor (AhR) is a ligand-activated transcription factor that promotes cell responses to small molecules derived from the diet, microorganisms, metabolism and pollutants. The AhR signal regulates many basic cellular processes, including cell cycle progression, adhesion, migration, apoptosis and cell proliferation. Many studies have shown that AhR is associated with chronic kidney disease (CKD) and its complications. This article reviews the current knowledge about the role of AhR in CKD, showing that AhR mediates CKD complications, including cardiovascular disease, anemia, bone disorders, cognitive dysfunction and malnutrition, and that it influences drug metabolism in individuals with CKD. AhR enhances the intestinal barrier function to reduce the harmful effects of uremic toxins. Therefore, understanding the complex roles of AhR during CKD is important to be able to target this transcription factor safely and effectively for CKD prevention and treatment.

## Introduction

Chronic kidney disease (CKD) has become an emerging global disease during the past decade. The all-age prevalence of CKD increased 29.3% worldwide from 1990 to 2017, involving 697.5 million cases in 2017 (representing a global prevalence of 9.1%) (Bikbov et al., 2020). CKD results in irreversible kidney structural and functional alterations that lead to end-stage renal disease, with patients ultimately requiring dialysis or renal transplantation.

Chronic kidney disease is characterized by the accumulation of a mixture of uremic toxins/solutes that persist even after standard dialysis (Vanholder and Glorieux, 2014; Sirich, 2017). These toxins are divided into three categories: small soluble compounds, middle molecules and protein-bound molecules (Duranton et al., 2012). Tryptophan-derived toxins are of particular interest because they exhibit cardiovascular toxicity and are ligands of the aryl hydrocarbon receptor (AhR) (Heath-Pagliuso et al., 1998; Schroeder et al., 2010). Multiple sensors detect changes in the cellular milieu to adapt to toxic environments and generate responses to molecular environment changes due to diet, symbiotic microbiome and host metabolism factors. One of these sensors is AhR, a ligand-activated transcription factor involved in various cellular processes, such as cell cycle, epithelial barrier function and neurological signaling, as well as responses to antioxidants and xenobiotics (Murray et al., 2014).

Aryl hydrocarbon receptor regulation and its role during CKD has gained interest as a potential source of biological processes that may guide the development of new therapeutic interventions. In this review, we summarize the current knowledge on the role of AhR in CKD and/or its complications, and we address the potential role of AhR as a therapeutic target.

## AhR Signaling and Its Ligands

### AhR Signaling Pathway

Aryl hydrocarbon receptor belongs to the periodic-AhR nuclear translocator-single-minded protein (PER-ARNT-SIM [PAS]) superfamily. The PAS domain senses both endogenous factors (such as cellular metabolites) and exogenous factors (such as environmental toxins) (McIntosh et al., 2010).

**FIGURE 1:**
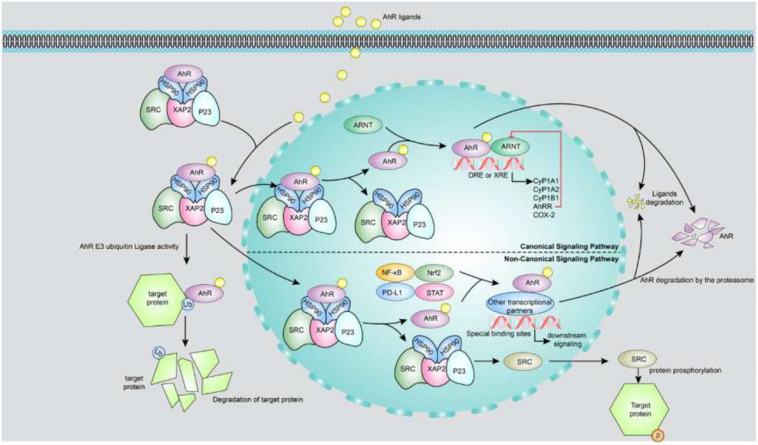
Canonical and non-canonical signaling pathways of AhR. The upper part of the figure is a canonical AhR signal pathway. Prior to AhR activation, AhR is inactive as part of a protein complex that consists of two molecules of HSP90, XAP2, p23 and the protein kinase SRC in the cytoplasm. Once a ligand binds to AhR, the molecule undergoes a conformational change to expose its N-terminal nuclear localization sequence, facilitating translocation of the liganded AhR protein complex into the nucleus. In the nucleus, AhR binds to ARNT through its PAS domain, forming a ligand-bound AhR-ARNT dimer. The dimer gets recruited to a DNA-specific sequence located within the promoters of target genes, to promote transcription of a wide variety of genes, such as Cyp1A1, CyP1B1, AhRR, and COX-2. AhR activity is strictly controlled by three different negative feedback loops, including: (i) degradation of AhR by proteasome, (ii) clearance of ligand by metabolic pathway, (iii) destruction of AhR/ARNT complex by AhRR. The lower part of figure shows the non-canonical AhR signaling: (i) AhR can also interact with NF-κB, Nrf2, PD-L1 and STAT proteins or other transcription factors to bind their specific binding sites and consequently modulate the activity and expression of their target genes. (ii) AhR functions as an E3 ubiquitin ligase to regulate the half-life of other transcription factors. (iii) AhR activation triggers phosphorylation cascades driven by SRC kinase when it releases from the AhR chaperone complex. AhR, aryl hydrocarbon receptor; AhRR, AhR repressor; ARNT, aryl hydrocarbon receptor nuclear translocator; COX-2, cyclooxygenase-2; Cyp1A1, cytochrome P450; family 1, member 1A; Cyp1A2, cytochrome P450; family 1, member 2A; Cyp1B1, cytochrome P450; family 1, sub family B; DRE or XRE, dioxin-or xenobiotic-responsive element; HSP90, heat-shock protein 90; NF-κB, nuclear factor-κB; Nrf2, nuclear factor-erythroid-2-related factor 2; P23, co-chaperon p23; PD-L1, programmed death ligand 1; STAT, signal transducer and activator of transcription; XAP2, X-associated protein 2.

As shown in Figure 1, there are two AhR signaling pathways. One is the xenobiotic-responsive element (XRE)-dependent control of gene expression by AhR, also called canonical AhR signaling. Prior to AhR activation, AhR is inactive as part of a protein complex that consists of two molecules of heat-shock protein 90, one X-associated protein 2 (also known as an AhR-interacting protein), one co-chaperon p23 and the protein kinase Sarcoma (SRC) in the cytoplasm. The AhR chaperone complex stabilizes AhR in the cytoplasm and keeps it in a conformation that has high affinity for its ligands (Pongratz et al., 1992). Once a ligand binds to AhR, the molecule undergoes a conformational change to expose its N-terminal nuclear localization sequence, facilitating translocation of the liganded AhR protein complex into the nucleus. In the nucleus, AhR binds to ARNT (also known as HIF-1β) through its PAS domain, forming a ligand-bound AhR-ARNT dimer. The dimer gets recruited to a DNA-specific sequence (referred to as a DRE or XRE for a dioxin- or xenobiotic-responsive element) located within the promoters of target genes, to promote transcription of a wide variety of genes, such as cytochrome P450, family 1, member 1A (Cyp1A1); cytochrome P450, family 1, member 2A; cytochrome P450, family 1, subfamily B; AhR repressor (AhRR) and cyclooxygenase-2 (Denison and Nagy, 2003). AhR-dependent gene transcription ends upon separation of the AhR-ARNT complex from the DRE. The N-terminal nuclear export sequence mediates AhR export from the nucleus into the cytoplasm, where ubiquitin-mediated AhR proteasome degradation occurs (Pollenz, 2002). The other AhR signaling pathway is non-canonical AhR signaling, which controls AhR gene expression through non-XRE DNA-response elements. AhR can also interact with additional transcription factors such as nuclear factor-κB (NF-κB), programmed death ligand 1, signal transducer and activator of transcription (STAT) and nuclear factor-erythroid-2-related factor 2 by binding to them and modulating the expression of their target genes through a non-genomic pathway that does not require ARNT (Kimura et al., 2008; Puga et al., 2009; Wakamatsu et al., 2018; Singh et al., 2019; Wang et al., 2019). Moreover, AhR functions as an E3 ubiquitin ligase, regulating the half-life of other transcription factors. For example, indoxyl sulphate (IS) directly activates AhR, extending the half-life of tissue factor (TF) by inhibiting its ubiquitination in vascular smooth muscle cells; TF is a crucial mediator of injury-related thrombosis (Shivanna et al., 2016). In addition, AhR activation triggers phosphorylation cascades driven by SRC kinase released from the AhR chaperone complex. For instance, SRC phosphorylates a broad variety of target proteins including indoleamine 2,3-dioxygenase 1 (IDO1) (Bessede et al., 2014), which promotes tryptophan transformation into kynurenine, a ligand of AhR.

### AhR Ligands

Aryl hydrocarbon receptor was discovered to be a receptor binding to 2,3,7,8-tetrachlorodibenzo-p-dioxin (TCDD) (also known as dioxin) with high affinity. The receptor can be hyperactivated in a sustained manner, resulting in numerous toxicological outcomes. In addition, many commercial and consumer products, vegetables, fruits, and spices have exhibited AhR activation potential (Jeuken et al., 2003; Zhao et al., 2013) and can facilitate the induction or repression of the expression of a range of genes, thereby regulating a diverse spectrum of biological and toxic effects. Environment pollutants and myriad endogenous molecules such as microbial metabolites (e.g., indolic solutes) and naturally occurring flavonoids (e.g., resveratrol and quercetin) are also AhR ligand candidates (Murray et al., 2014; Gutierrez-Vazquez and Quintana, 2018; Rothhammer and Quintana, 2019; Table 1).

**TABLE 1:** exogenous and endogenous AhR ligands.

Ligand		Biochemical pathway
Xenobiotic	2,3,7,8-tetrachlorodibenzo-p-dioxin (TCDD)	Halogenated aromatic hydrocarbons
	3-methylcholanthrene	
	Benzanthracenes	
	Benzoflavones	
	Benzo[a]pyrene	
	Biphenyls	
	Dibenzofurans	
	Polyaromatic hydrocarbons	
	Leflutamide	Pharmaceuticals
	Tranilast	
	Omeprazole	
	6,2′,4′,-trimethoxyflavone	Compound from chemical library
	CH-223191	
	GNF351	
	StemRegenin 1 (SR1)	
	Indirubin	Phytochemicals from plants
	Indigo	
Diet	Galangin	Flavonoids
	Quercetin	
	Kaempferol	
	Resveratrol	Polyphenol
	Indole-3-acetonitrile	Dietary metabolite of cruciferous vegetables
	Indole-3-carbinole	
	Indolo[3,2-b]carbazole (ICZ)	
	3,3′-diindolylmethane (DIM)	
Tryptophan metabolites	3-Methylindole (Skatole)	Microbiota metabolism
	Indole-3-acetaldehyde	
	Indole-3 acetic acid (IAA)	
	Indole-3-aldehyde (IAld)	
	Tryptamine (TA)	
	5-hydroxy-tryptophan (5HTP)	Host metabolism
	Cinnabarinic acid (CA)	
	Kynurenic acid (KA)	
	Kynurenine (Kyn)	
	Xanthurenic acid	
	Indoxyl sulfate (IS)	Microbiota and host metabolism
	6-formylindolo[3,2-b]carbazole (FICZ)	Photo-oxidation
Other metabolites	Bilirubin	Haem metabolism
	Biliverdin	
	Lipoxin 4A	Arachidonic Acid Metabolism
	Prostaglandin PGG2	
	Hydroxyeicosatrienoic acid ([12(R)-HETE])	
	1,4-dihydroxy-2-naphtoic acid	Microbiota metabolism
	3-methylindole	
	7-ketocholesterol	
	Indirubin	
	Malassezin	
	Trypthantrin	

For patients with CKD, the most important AhR ligands are uremic toxins, especially those derived from tryptophan metabolism. This essential aromatic amino acid is a biosynthetic precursor of a large number of microbial and host metabolites (Alkhalaf and Ryan, 2015). Tryptophan metabolism follows three major pathways in the gastrointestinal tract: (1) the indolic pathway in intestinal microorganisms that directly transforms tryptophan into several AhR ligand molecules, such as indole-3-acid-acetic (IAA), indole-3-aldehyde, indole-3-acetaldehyde, and indole-3-propionic acid (Zelante et al., 2013; Hubbard et al., 2015; Alexeev et al., 2018); (2) the kynurenine pathway via the rate-limiting enzyme IDO1 that produces kynurenine and downstream products such as quinolinic acid and kynurenic acid (Clarke et al., 2012; Cervenka et al., 2017; Kennedy et al., 2017) and (3) the serotonin pathway in enterochromaffin cells that involves tryptophan hydroxylase-1 (Yano et al., 2015). Some tryptophan metabolites such as IS, IAA and kynurenine represent both important uremic toxins and potent AhR ligands. We will summarize the associations between AhR and uremic toxins in CKD below.

## Activated AhR Aggravates Renal Damage

Patients with CKD are exposed to a variety of uremic toxins, which are agonists of AhR. Early Lu et al. (2006) provided evidence for a positive correlation between renal AhR expression and CKD severity, suggesting that AhR activation has a pathogenic effect in both male and female rats that have undergone nephrectomy. Compared with healthy controls, CKD patients and 5/6 of nephrectomy mice exhibited increased serum AhR-activating potential, which was strongly correlated with IS concentration and estimated glomerular filtration rate, and which upregulated expression of the AhR target genes AhRR and Cyp1A1 (Dou et al., 2018). Kim et al. (2020) reported higher AhR transactivating (AhRT) activity in patients with CKD than in controls, and they showed that peritoneal dialysis treatment was more effective at reducing this AhRT activity than haemodialysis.

Aryl hydrocarbon receptor signaling regulates many fundamental cellular processes, such as cell cycle progression, apoptosis and cell proliferation by regulating P53, FasR, Bcl-2 and cell cycle kinases (Zaher et al., 1998; Mohammadi et al., 2017). Exposure to adenine and IS increases AhR activity in the periglomerular region as well as in proximal and distal renal tubules, leading to renal fibrosis (Walker et al., 2020). AhR activated by IS induces podocyte injury, progressive glomerular damage and a pro-inflammatory phenotype *in vitro* and *in vivo* (Ichii et al., 2014). IS downregulates the expression of the Mas receptor, which is associated with the inhibition of the renin-angiotensin system, via the organic anion transporter 3/AhR/STAT3 pathway to upregulate TGF-β in proximal tubular cells, thereby aggravating CKD (Ng et al., 2014). A possible connection between AhR and renal fibrosis has been further supported by the association between indole solutes and renal fibrosis (Mutsaers et al., 2015b), which may be mediated by AhR signaling.

In patients with diabetes mellitus, AhRT activity has been positively correlated with the progression of diabetic nephropathy (DN) and the severity of renal insufficiency (Kim et al., 2013). Lee et al. (2016) investigated the role of AhR in the pathophysiological processes of DN using AhR-knockout and pharmacological-inhibitor α-naphthoflavone models, and they found that AhR mediated renal oxidative stress in a diabetic mouse model, resulting in macrophage infiltration, extracellular matrix accumulation and mesangial cell activation.

Translocation of microbial antigens due to gut barrier breakdown has been associated with gut microbiota and the presence of systemic lupus erythematosus (SLE) and lupus nephritis in animal and human studies. Clinical studies have shown reduced gut microbiota diversity in patients with SLE, and gut-derived pathobionts including *Enterococcus gallinarum* have been detected in the liver (Manfredo Vieira et al., 2018). Animal studies have shown that gut-derived *E. gallinarum* can reach the liver and induce the development of lupus autoantibodies, partly via activation of the AhR/CYP1A1 pathway to trigger Th17 and T follicular helper cell activation, crucial steps for the production of systemic autoantibodies including anti-dsDNA antibodies (Manfredo Vieira et al., 2018).

Therefore, AhR may be a therapeutic target in CKD. In fact, resveratrol, a natural AhR antagonist, inhibits proteinuria, hypoalbuminemia and hyperlipidemia in nephritic rats (Nihei et al., 2001). Atrazine exposure has been shown to cause environmental nephrosis via direct kidney injury, whereas supplementary lycopenes provide significant protection against atrazine-induced renal injury and environmental nephrosis via their inhibition of the activation of AhR and the expression of cytochrome P450 isoforms (Xia et al., 2018). On the other hand, ARNT, a transcriptional co-activator of AhR, has been implicated in renal fibrosis and is sought as a therapeutic target in CKD; strong experimental support exists for a mechanistic model in which FK506 induces renoprotection (Tampe et al., 2018; Haase, 2019).

## AhR Activation Mediates Various Complications in Individuals With CKD

### AhR and Cardiovascular Disease

Cardiovascular disease is a major cause of mortality in patients with CKD. AhR is an important receptor for various toxins and can activate pro-thrombotic and pro-inflammatory pathways; thus, there is a close association between the accumulation of uremic toxins and the cardiovascular complications of CKD (Vanholder et al., 2016). In fact, even under non-uremic conditions, AhR activation is associated with increased cardiovascular risks (Sallee et al., 2014). Accordingly, individuals exposed to AhR agonists are prone to increased cardiovascular risks (Humblet et al., 2008). For example, individuals exposed to the TCDD compound in Agent Orange during the Vietnam War have been shown to have associated cardiovascular complications ranging from coronary heart disease to stroke (Lowenstein, 2014).

The accumulation of uremic toxins has effects similar to those of AhR antagonists, making patients with CKD more prone to cardiovascular disease through a series of possible mechanisms. Ito et al. (2016) proved that AhR activated by IS can stimulate the transcriptional activity of activator protein-1 and induce the expression of E-selectin in vascular endothelial cells, thereby increasing the level of vascular inflammation and leukocyte adhesion. Assefa et al. (2019) showed that IS-activated AhR upregulates the activity of SRC to mediate the activity of vascular endothelial cadherin, thereby increasing endothelium permeability and promoting atherosclerosis. In human hepatoma HepG2 cells, IS-induced AhR activation inhibits the expression of fetuin-A, a liver-derived circulating protein that effectively suppresses calcification. Therefore, IS-activated AhR may increase the incidence of vascular calcification and cardiovascular mortality (Ochi et al., 2015). In human umbilical vein endothelial cells, AhR also mediates IS-induced cellular senescence (Koizumi et al., 2014; Eckers et al., 2016) and stimulates the expression of monocyte chemoattractant protein-1, a chemokine recruiting monocytes from the bloodstream into the subendothelial space to participate in the early stages of atherosclerosis (Watanabe et al., 2013). AhR further plays a key role during platelet production (Lindsey and Papoutsakis, 2011; Strassel et al., 2016) and function (Lindsey et al., 2014). AhR agonists enhance platelet aggregation by activating the AhR/p38MAPK pathway in platelets (Pombo et al., 2015). In addition, activated AhR increases the expression and activity of TF to induce a procoagulant state in vascular smooth muscle and endothelial cells (Gondouin et al., 2013; Shivanna et al., 2016); moreover, it enhances the activation of the pro-inflammatory enzyme cyclooxygenase-2 in endothelial cells (Dou et al., 2015). Vascular dysfunction caused by AhR activation may lead to atherosclerotic thrombosis, thus increasing the risk of myocardial infarction, stroke and peripheral artery disease in patients with CKD (Vanholder et al., 2016).

These summarized mechanisms of AhR activation in CKD support efforts to target AhR and limit the detrimental effects of uremic toxins on vascular outcomes. AhR has been considered a therapeutic target for improving the management of the cardiovascular complications of CKD (Shivanna et al., 2016). In Gondouin’s study, IS and IAA elevated the production of TF in peripheral blood mononuclear and endothelial cells after AhR activation, generating a “dioxin-like” effect (Gondouin et al., 2013). Addi et al. (2019) confirmed that CH223191, an AhR-specific antagonist, could decrease the expression of TF in a process mediated by IAA in human endothelial cells. Meanwhile, Assefa et al. (2019) showed that resveratrol inhibits the activation of SRC and downregulates the activity and permeability of the vascular endothelium by blocking the IS/AhR pathway, thereby provoking protective effects.

### AhR and Anemia

Renal anemia is common in CKD patients and is an important risk factor for CKD progression and mortality (Mohanram et al., 2004; Levin et al., 2006). The prevalent cause of renal anemia is the insufficient production of erythropoietin (EPO) that results from renal interstitial fibrosis (Souma et al., 2015). The transcription of EPO mRNA is mediated by a hypoxia-inducible transcription factor (HIF) formed by HIF-αs (including HIF-1α and HIF-2α) and ARNT. The expression of HIF-αs is strictly regulated by the concentration of cellular oxygen molecules. HIF-α degradation is inhibited under hypoxic conditions, and the resulting nuclear accumulation of HIF-α and its dimerization with ARNT promotes the expression of target genes including EPO. Because both HIF and AhR are members of the PAS-domain protein family and contain basic helix loops, AhR competes with HIF-α subunits to combine with ARNT and form heterodimeric transcription factor complexes in the nucleus (Soshilov and Denison, 2008). In individuals with CKD, uremic toxins elevate AhR levels and may provoke the accumulation of AhR-ARNT complexes while inhibiting the formation of HIF-α-ARNT complexes in the nucleus, leading to abnormal intracellular HIF signal transduction and the transition of renal interstitial EPO-producing tissues into a pathological fibrotic state associated with impaired EPO production capacity (Souma et al., 2013, 2016; Mascanfroni et al., 2015).

Asai et al. (2016), first reported that IS suppresses hypoxic HIF activation and inhibits the production of EPO in rats and HepG2 cells, confirming the presence of IS-impaired HIF signaling due to AhR activation in renal anemia. Later, the same researchers found that indole glucuronic acid inhibits the expression of HIF-dependent EPO by activating AhR, whereas the drug antagonist CH-223191 blocks AhR and abolishes the inhibiting effect of indole glucuronide on HIF (Asai et al., 2018).

Renal anemia has the same signs as iron deficiency anemia, which is caused by impaired iron utilization. Hepcidin, a liver-secreted protein, is essential for iron metabolism regulation (Park et al., 2001); it induces the internalization and degradation of a cellular iron exporter called ferroportin that regulates intracellular iron efflux (Nemeth et al., 2004). Therefore, the increased hepcidin levels associated with CKD cause duodenal iron absorption disorder and intracellular iron retention, which aggravate the functional iron deficiency state in CKD. Hamano et al. (2018) showed that the expression of hepcidin is increased by IS in a dose-dependent manner and suppressed by AhR silencing in HepG2 cells.

### AhR and Bone Disorders

Chronic kidney disease mineral and bone disorders are generally characterized by changes in bone metabolism. These systemic disorders are common in patients with CKD and are characterized by calcium and phosphorus abnormalities, vitamin D deficiency, vascular calcification, secondary hyperparathyroidism and bone abnormalities (Kidney Disease: Improving Global Outcomes (KDIGO) CKD-MBD Update Work Group, 2017).

Vidal et al. (2015) observed that the amount of tryptophan degraded through the kynurenine pathway increases significantly during the formation of osteoblasts and that osteoblast formation is inhibited when IDO1, the enzyme that stimulates the conversion of tryptophan to kynurenine, is blocked. Data published by Refaey et al. (2017) showed that intragastric or intraperitoneal kynurenine administration impairs osteoblast differentiation and increases osteoclast absorption to accelerate age-related bone loss.

Aryl hydrocarbon receptor is a cytoplasmic receptor of several low-molecular-weight exogenous and endogenous molecules that can control bone homeostasis by regulating osteoclast differentiation through the RANKL/AhR/c-Fos signaling axis (Izawa et al., 2016). Exposure to TCDD (the most widely known exogenous AhR ligand) weakens the mechanical strength of bone, hardens the bone matrix and makes the cortical bone thinner and looser in wild-type mice, but these effects are diminished in AhR-knockout mice (Herlin et al., 2013). In addition, AhR-knockout mice exhibit decreased bone resorption and increased bone mass. Kynurenine has been identified as an endogenous AhR agonist (Opitz et al., 2011). Kalaska et al. (2017) observed that the level of peripheral kynurenine is negatively correlated with bone biomechanical variables, bone geometric variables and bone mineral density, and that high peripheral kynurenine levels cause pathological changes in bone microstructure and strength through the AhR pathway in CKD rats that had undergone subtotal nephrectomy.

### AhR and Cognitive Dysfunction

The severity of cognitive impairment varies from mild cognitive impairment to severe dementia and is characterized by the loss of independence in daily activities (American Psychiatric Association, 2013). Cognitive impairment may already be present during the early stages of CKD, and it becomes more severe as CKD progresses (Bugnicourt et al., 2013; Brodski et al., 2019). In a 6-year longitudinal study conducted in the general population, the impact of CKD on the risk of mild cognitive impairment and dementia exceeded the impacts of long-term use of anti-anxiety drugs, stroke or genetic factors (Lipnicki et al., 2017).

Factors such as white matter damage, asymptomatic infarction and microhaemorrhages may be involved in cognitive impairment (Bugnicourt et al., 2013), but vascular injury is the basic factor underlying all these pathological manifestations. AhR is thought to play an essential role in the vascular dysfunctions seen in patients with cardiovascular disease.

Aryl hydrocarbon receptor is expressed in the cerebral cortex, hippocampus, cerebellum and other sites of the brain (Lin et al., 2008), and the receptor is thought to be associated with cognitive dysfunction caused by oxidative stress or excitotoxicity (Kim and Yang, 2005; Williamson et al., 2005). Walker et al. (2020) found that activated AhR in brain microvascular endothelial cells in CKD models induced by adenine and IS treatment (in an uremic environment) plays a role in the biology and pathology of central nervous system endothelium. An increase in the production of reactive oxygen species (ROS) aggravates inflammation in patients with neurodegenerative diseases (Hsieh and Yang, 2013). In Adesso’s study, increased expression of AhR in primary astrocytes and mixed glial cells was found after IS treatment; in addition, ROS production was significantly reduced after the addition of an AhR inhibitor, showing that the AhR/ROS pathway is activated by IS in the central nervous system of patients with CKD (Adesso et al., 2018).

### AhR and Protein Energy Waste (PEW)

Protein energy waste (PEW) is a common complication with adverse consequences in patients with CKD. PEW is characterized by the gradual loss of protein and energy fuels stored in the body (i.e., body muscle and fat mass) (Ikizler et al., 2013). The loss of body muscle mass is caused by many factors, including insufficient energy and protein intake, protein loss in the urine and chronic inflammation (Carrero et al., 2013).

Enoki et al. (2016) demonstrated that IS activates the AhR/NADPH oxidase/NF-κB pathway, leading to increased ROS production, which then triggers the production of inflammatory markers and increases the expression of atrogin-1, myostatin, IL-6 and TNF-α to cause muscle atrophy. AhR inhibitors (e.g., CH-223191) and AhR-siRNA significantly inhibit the production of inflammatory cytokines (ROS and atrophy-related genes in C2C12 myoblasts), suggesting that the activation of AhR in cells is associated with muscle atrophy (Enoki et al., 2016).

### AhR and Drug Metabolism

Patients with CKD have high rates of complications and comorbidities. Thus, they require the prescription of numerous medications. However, the pharmacokinetics of drugs in these patients are changed by the uremic environment, making drug dosage adjustments challenging and drug-related adverse events frequent. However, the cellular and molecular mechanisms involved remain unclear. There are more than 200 P450 proteins, and AhR signaling regulates three of them (CYP1B1, CYP1A1, and CYP1A2) (Fujii-Kuriyama and Mimura, 2005). Mutsaers et al. (2015a) showed that uremic toxins reduce the activity of some drug transporters. Santana et al. (2018) proved that IS affects the blood levels of cyclosporine by regulating transport proteins such as P-glycoprotein through AhR activation. Therefore, AhR seems to be associated with the altered drug clearance seen in CKD. The regulation of AhR activity may be a target for improving drug clearance in patients with CKD.

## AhR Enhances Intestinal Barrier Function

**FIGURE 2:**
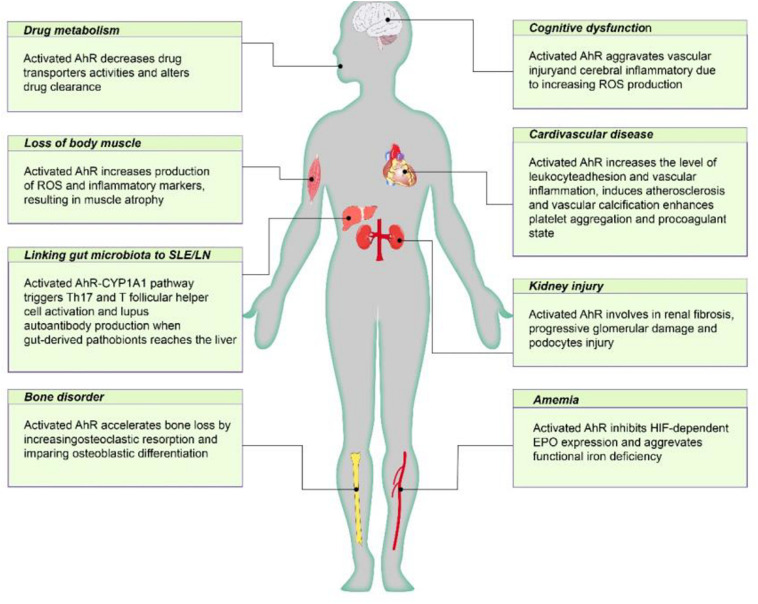
The negative effect of AhR poses on CKD individuals. Active AhR aggravates renal damage, mediates various CKD complications, including cardiovascular disease, anemia, bone disorders, cognitive dysfunction, protein energy wasting, and influences drug metabolism. AhR, aryl hydrocarbon receptor; Cyp1A1, cytochrome P450; family 1, member 1A; EPO, erythropoietin; HIF, hypoxia inducible factor; ROS, reactive oxygen species.

**FIGURE 3:**
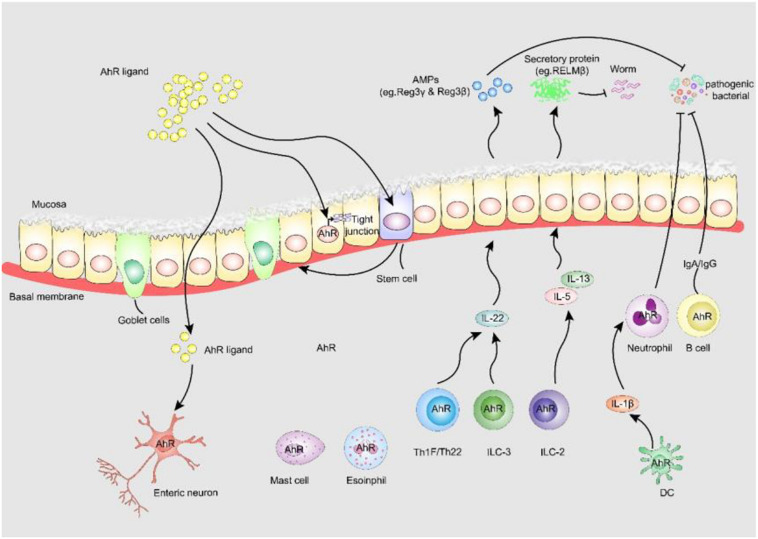
AhR enhances intestinal barrier function. (i) AhR is identified as fulcrum of an enteric nervous system-specific surveillance pathway that regulates intestinal peristalsis in response to microbial colonization, maintaining gut homeostasis and host defense. (ii) AHR in ISC stabilizes gut epithelial barrier function and controls the regeneration of gut ILEs. (iii) AhR upregulates epithelial tight junction protein the tight junction between IECs. (iv) AhR is expressed by many adaptive and innate immune cells in the lamina propria, including macrophages, mast cells, eosinophils, DCs, B cells and T cells, AhR signaling is pivotal to the regulation of mucosal intestinal immune responses. AhR, aryl hydrocarbon receptor; AMPs, anti-microbial peptides; RELM-β, resistin-like molecule β; Reg3-γ, regenerating Islet Derived Protein-γ; Reg3-β, regenerating Islet Derived Protein-β.

As discussed above, AhR mediates renal damage and CKD complications and seems to be harmful in CKD patients (Figure 2). However, AhR is thought to play an active role in intestinal homeostasis (Figure 3). AhR was found to be expressed in almost all colonic muscularis layers, whereas it was absent from the duodenal and jejunal muscular layers. At the same time, only relatively weak signals were detected in ileum terminal neurons, suggesting that AhR expression in intestinal neurons is synchronous with the intestinal microbial load (Obata et al., 2020). AhR influences different aspects of intestinal barrier function. First, AhR is considered important for specific monitoring pathways of the intestinal nervous system that regulate intestinal responses to colonization, the maintenance of intestinal functions and host defenses (Obata et al., 2020). Therefore, pharmacological or dietary interventions that modulate AhR activity in the cellular circuitry to control intestinal peristalsis offer a realistic strategy for the management of conditions associated with gut dysmotility, including the constipation due to slow colonic transit common in patients with CKD.

In addition to the role of AhR in neurogenic motility, AhR-dependent transcriptional procedures are also essential for the barrier function of intestinal epithelial cells (IECs) (Metidji et al., 2018; Rothhammer and Quintana, 2019). AhR in intestinal stem cells stabilizes intestinal epithelial barrier function and controls the regeneration of IECs. Lack of IEC-specific AhR results in the failure to protect against *Citrobacter rodentium* infection due to reduced quantities of mucus-producing goblet cells (Metidji et al., 2018). AhR activation by dietary ligands may restore barrier homeostasis and protect the stem cell niche. Furthermore, AhR can upregulate epithelial tight junctions. 6-formylindolo[3,2-b]carbazole (FICZ), a high-affinity endogenous ligand of AhR, prevents the decrease of ZO-1, occludin and claudin-1 to modulate intestinal epithelial barrier function in dextran sulfate sodium-induced IECs (Yu et al., 2018). A metabolite of *Lactobacillus*, indole-3-aldehyde, stimulates lamina propria lymphocytes to secrete AhR/IL-22 pathway products and induces the phosphorylation of STAT3 to accelerate intestinal epithelial proliferation, thereby healing damaged intestinal mucosae (Hou et al., 2018).

Furthermore, the expression of AhR by many adaptive and innate immune cells in the lamina propria, including macrophages, mast cells, eosinophils, dendritic cells (DCs), B cells and T cells, suggests that AhR signaling is essential for the regulation of intestinal mucosal immune responses. DCs are prototypic targets of environmental exposure and endogenous AhR ligands (Quintana et al., 2015). Macrophages and DCs produce high levels of IL-1β, which promotes neutrophil recruitment and pathogen elimination. AhR is also essential for the function of innate lymphoid cells (ILCs) and Th17/Th22 cells that control symbiosis and pathogens through IL-22-pSTAT3 (Wang et al., 2014; Piccinni et al., 2019) and mediate the production of antimicrobial peptides by IECs (Sonnenberg et al., 2011). Antimicrobial peptides can also participate in colonization resistance against pathogens by shaping the microbiota, which compete with pathogens for nutrients. Genetic AhR ablation enhances gut ILC2 function during anti-helminth immunity, and cell-specific activation of AhR enhances gut ILC3 function for anti-bacterial immunity, suggesting the central role of AhR expression for gut adaptation and ILC2-ILC3 balance (Li et al., 2018).

Since Meijers proposed the existence of a gut-kidney axis (Meijers and Evenepoel, 2011), some studies have discovered that damage to the gut barrier function induced by intestinal dysbiosis in CKD allows the translocation of endotoxin and bacterial metabolites into the circulation, which causes uremic toxicity, inflammation, CKD progression and cardiovascular disease (Meijers et al., 2019). As our understanding of the association between the gut and the kidney continues to grow, various therapies targeting the colonic microenvironment in CKD have been developed, such as regulating gut microbiota, blocking lipopolysaccharides, attenuating inflammation or targeting uremic toxin end-products of microbial fermentation (Ramezani and Raj, 2014). Although few therapies target AhR to improve intestinal barrier function in patients with CKD, the potential exists to uncover many novel avenues to delay CKD progress; for example, baicalein, a flavonoid from *Scutellaria baicalensis* used in Chinese herbal medicine, induces regulatory T-cell differentiation through AhR and enhances intestinal barrier function through its regulation of tight junctions in a mouse model of food allergies (Bae et al., 2016).

## Concluding Remarks

Initial studies on AhR were focused on its role as a chemical sensor signaling molecule responding to environment pollutants, but the range of subjects researched has gradually expanded to include diseases such as cancer and cardiovascular and kidney disorders. AhR mediates CKD progression and its complications, including cardiovascular disease, anemia, bone disorders, cognitive dysfunction and malnutrition, and it affects the drug metabolism of individuals with CKD. On the other hand, AhR enhances intestinal barrier function and reduces the harmful effects of uremic toxins. Therefore, whether AhR helps patients with CKD to deal with the pathological changes they face or increases their risk for pathologic complications remains unclear. Based on the available data, we could not draw a definite conclusion about a positive role for AhR in intestinal homeostasis, but we know that it mediates renal damage and CKD complications outside the gastrointestinal tract. AhR-mediated detoxification mechanisms stimulate the clearance of endobiotics and xenobiotics by the kidney. AhR may help individuals to survive in changing environments or promote self-recovery during the early stages of disease, but once the normal physiological or protective responses of the AhR pathway are interrupted, the severity of disease increases. Regulating the AhR pathway is without a doubt an attractive treatment strategy for CKD. However, more studies are needed to overcome the interpretation challenges of the current data.

First, AhR ligands have different sources and structures, present diverse receptor affinities and produce different downstream signal transduction effects. Whether a certain type of AhR ligand is beneficial or detrimental under a specific situation needs to be determined. New metabolomics methods are helping researchers to discover, isolate and identify new exogenous and endogenous AhR ligands. An effective AhR ligand may not be able to play a “one size fits all” role throughout the entire CKD treatment process. In fact, even in a single model, an AhR-ligand combination may be temporarily effective in terms of protecting the body, but as the disease progresses, the interaction may turn harmful, or vice versa. In view of the conflicting observations on whether AhR promotes or inhibits inflammation, identifying and developing specific high-affinity AhR ligands to slow down the development of CKD and its complications is difficult. The ligands need to display targeted absorption, distribution, metabolism and excretion characteristics.

Second, a food-as-medicine approach could be used as a strategy based on bioactive nutrients to target the AhR pathway in patients with CKD. Natural bioactive compounds, including galangin, quercetin, kaempferol, resveratrol and dietary metabolites of cruciferous vegetables, are AhR ligands and potential nutritional therapeutic agents that may modulate the expression of pro-inflammatory factors. Suitable diet-derived AhR ligands should be considered during the dietary management of nutrients in patients with CKD, to put the ancient adage of “let food be thy medicine” into practice. Moreover, bioactive nutrients such as flavonoids and polyphenol compounds are widely distributed in natural products such as medicinal plants, tea, fruits and vegetables. In view of the important roles of the above-mentioned compounds in regulating AhR activity, further studies should address the regulation of AhR activity by natural compounds, to reveal their potential as therapeutic agents for CKD and demonstrate their molecular mechanisms.

Third, inducing the organism to produce appropriate AhR ligands is also a potential therapeutic strategy. Both indole-3-pyruvate (IPYA) and IS are derived from gut bacterial metabolism of the essential amino acid tryptophan. IPYA depends on AhR; it protects the intestine against excessive permeability by maintaining the integrity of apical junctional complexes and the associated actin regulatory proteins (Mafra et al., 2020), whereas IS induces intestinal barrier injury by impairing IRF1/DRP1 axis-mediated mitophagy (Huang et al., 2020). Therefore, inhibiting or promoting certain enzyme activities or regulating the intestinal flora for the metabolism of some key substances may induce the production of AhR ligands that are beneficial to the body.

Finally, although AhR is a promising target for clinical applications, most of the knowledge about its physiological and pathological functions in CKD comes from animal models, and the translation of experimental results into clinical applications for patients with CKD is difficult. Therefore, further research is needed to better understand the complex roles AhR plays in individuals with CKD, so as to be able to use it as a safe and effective target for CKD prevention and treatment.

## Author Contributions

XL: conceptualization. YM, ZL, CJ, and XL: literature search. YM, ZL, and XL: writing, formatting, and figures. LW, CZ, and XL: review and editing. All authors read and approved the final manuscript.

## Conflict of Interest

The authors declare that the research was conducted in the absence of any commercial or financial relationships that could be construed as a potential conflict of interest.

## References

[B1] Addi T. Poitevin S. McKay N. El Mecherfi K. E. Kheroua O. Jourde-Chiche N. (2019). Mechanisms of tissue factor induction by the uremic toxin indole-3 acetic acid through aryl hydrocarbon receptor/nuclear factor-kappa B signaling pathway in human endothelial cells. *Arch. Toxicol.* 93 121–136. 10.1007/s00204-018-2328-3 30324315

[B2] Adesso S. Paterniti I. Cuzzocrea S. Fujioka M. Autore G. Magnus T. (2018). AST-120 reduces neuroinflammation induced by Indoxyl sulfate in Glial cells. *J. Clin. Med.* 7:365. 10.3390/jcm7100365 30336612 PMC6210605

[B3] Alexeev E. E. Lanis J. M. Kao D. J. Campbell E. L. Kelly C. J. Battista K. D. (2018). Microbiota-derived indole metabolites promote human and Murine intestinal homeostasis through regulation of interleukin-10 receptor. *Am. J. Pathol.* 188 1183–1194. 10.1016/j.ajpath.2018.01.011 29454749 PMC5906738

[B4] Alkhalaf L. M. Ryan K. S. (2015). Biosynthetic manipulation of tryptophan in bacteria: pathways and mechanisms. *Chem. Biol.* 22 317–328. 10.1016/j.chembiol.2015.02.005 25794436

[B5] American Psychiatric Association (2013). *Diagnostic and Statistical Manual of Mental Disorders (DSM-5).* Washington, DC: APA.

[B6] Asai H. Hirata J. Hirano A. Hirai K. Seki S. Watanabe-Akanuma M. (2016). Activation of aryl hydrocarbon receptor mediates suppression of hypoxia-inducible factor-dependent erythropoietin expression by indoxyl sulfate. *Am. J. Physiol. Cell Physiol.* 310 C142–C150. 26561638 10.1152/ajpcell.00172.2015

[B7] Asai H. Hirata J. Watanabe-Akanuma M. (2018). Indoxyl glucuronide, a protein-bound uremic toxin, inhibits hypoxia-inducible factordependent erythropoietin expression through activation of aryl hydrocarbon receptor. *Biochem. Biophys. Res. Commun.* 504 538–544. 10.1016/j.bbrc.2018.09.018 30205954

[B8] Assefa E. G. Yan Q. Gezahegn S. B. Salissou M. T. M. He S. Wu N. (2019). Role of resveratrol on indoxyl sulfate-induced endothelial hyperpermeability via Aryl hydrocarbon receptor (AHR)/Src-dependent pathway. *Oxid. Med. Cell. Longev.* 2019:5847040. 10.1155/2019/5847040 PMC6900952 31885805

[B9] Bae M. J. Shin H. S. See H. J. Jung S. Y. Kwon D. A. Shon D. H. (2016). Baicalein induces CD4(+)Foxp3(+) T cells and enhances intestinal barrier function in a mouse model of food allergy. *Sci. Rep.* 6: 32225. 10.1038/srep32225 PMC4999817 27561877

[B10] Bessede A. Gargaro M. Pallotta M. T. Matino D. Servillo G. Brunacci C. (2014). Aryl hydrocarbon receptor control of a disease tolerance defence pathway. *Nature* 511 184–190. 24930766 10.1038/nature13323 PMC4098076

[B11] Bikbov B. Purcell C. A. Levey A. S. Smith M. Abdoli A. Abebe M. (2020). Global, regional, and national burden of chronic kidney disease, 1990–2017: a systematic analysis for the Global Burden of Disease Study 2017. *Lancet* 395 709–733. 32061315 10.1016/S0140-6736(20)30045-3 PMC7049905

[B12] Brodski J. Rossell S. L. Castle D. J. Tan E. J. (2019). A systematic review of cognitive impairments associated with kidney failure in adults before natural age-related changes. *J. Int. Neuropsychol. Soc.* 25 101–114. 10.1017/s1355617718000917 30463631

[B13] Bugnicourt J. M. Godefroy O. Chillon J. M. Choukroun G. Massy Z. A. (2013). Cognitive disorders and dementia in CKD: the neglected kidney-brain axis. *J. Am. Soc. Nephrol.* 24 353–363. 10.1681/asn.2012050536 23291474

[B14] Carrero J. J. Stenvinkel P. Cuppari L. Ikizler T. A. Kalantar-Zadeh K. Kaysen G. (2013). Etiology of the protein-energy wasting syndrome in chronic kidney disease: a consensus statement from the International Society of Renal Nutrition and Metabolism (ISRNM). *J. Ren. Nutr.* 23 77–90. 10.1053/j.jrn.2013.01.001 23428357

[B15] Cervenka I. Agudelo L. Z. Ruas J. L. (2017). Kynurenines: Tryptophan’s metabolites in exercise, inflammation, and mental health. *Science* 357:eaaf9794. 10.1126/science.aaf9794 28751584

[B16] Clarke G. McKernan D. P. Gaszner G. Quigley E. M. Cryan J. F. Dinan T. G. (2012). A distinct profile of tryptophan metabolism along the kynurenine pathway downstream of toll-like receptor activation in irritable bowel syndrome. *Front. Pharmacol.* 3:90. 10.3389/fphar.2012.00090 22661947 PMC3357104

[B17] Denison M. S. Nagy S. R. (2003). Activation of the aryl hydrocarbon receptor by structurally diverse exogenous and endogenous chemicals. *Annu. Rev. Pharmacol. Toxicol.* 43 309–334. 12540743 10.1146/annurev.pharmtox.43.100901.135828

[B18] Dou L. Poitevin S. Sallee M. Addi T. Gondouin B. McKay N. (2018). Aryl hydrocarbon receptor is activated in patients and mice with chronic kidney disease. *Kidney Int.* 93 986–999. 10.1016/j.kint.2017.11.010 29395338

[B19] Dou L. Sallee M. Cerini C. Poitevin S. Gondouin B. Jourde-Chiche N. (2015). The cardiovascular effect of the uremic solute indole-3 acetic acid. *J. Am. Soc. Nephrol.* 26 876–887. 10.1681/asn.2013121283 25145928 PMC4378098

[B20] Duranton F. Cohen G. De Smet R. Rodriguez M. Jankowski J. Vanholder R. (2012). Normal and pathologic concentrations of uremic toxins. *J. Am. Soc. Nephrol.* 23 1258–1270. 10.1681/asn.2011121175 22626821 PMC3380651

[B21] Eckers A. Jakob S. Heiss C. Haarmann-Stemmann T. Goy C. Brinkmann V. (2016). The aryl hydrocarbon receptor promotes aging phenotypes across species. *Sci. Rep.* 6:19618. 10.1038/srep19618 PMC4726214 26790370

[B22] Enoki Y. Watanabe H. Arake R. Sugimoto R. Imafuku T. Tominaga Y. (2016). Indoxyl sulfate potentiates skeletal muscle atrophy by inducing the oxidative stress-mediated expression of myostatin and atrogin-1. *Sci. Rep.* 6:32084. 10.1038/srep32084 PMC4994088 27549031

[B23] Fujii-Kuriyama Y. Mimura J. (2005). Molecular mechanisms of AhR functions in the regulation of cytochrome P450 genes. *Biochem. Biophys. Res. Commun.* 338 311–317. 10.1016/j.bbrc.2005.08.162 16153594

[B24] Gondouin B. Cerini C. Dou L. Sallee M. Duval-Sabatier A. Pletinck A. (2013). Indolic uremic solutes increase tissue factor production in endothelial cells by the aryl hydrocarbon receptor pathway. *Kidney Int.* 84 733–744. 10.1038/ki.2013.133 23636172

[B25] Gutierrez-Vazquez C. Quintana F. J. (2018). Regulation of the immune response by the Aryl hydrocarbon receptor. *Immunity* 48 19–33. 10.1016/j.immuni.2017.12.012 29343438 PMC5777317

[B26] Haase V. H. (2019). ARNT as a novel antifibrotic target in CKD. *Am. J. Kidney Dis.* 73 281–284. 10.1053/j.ajkd.2018.08.009 30343956 PMC6348016

[B27] Hamano H. Ikeda Y. Watanabe H. Horinouchi Y. Izawa-Ishizawa Y. Imanishi M. (2018). The uremic toxin indoxyl sulfate interferes with iron metabolism by regulating hepcidin in chronic kidney disease. *Nephrol. Dial. Transplant.* 33 586–597. 10.1093/ndt/gfx252 28992067

[B28] Heath-Pagliuso S. Rogers W. J. Tullis K. Seidel S. D. Cenijn P. H. Brouwer A. (1998). Activation of the Ah receptor by tryptophan and tryptophan metabolites. *Biochemistry* 37 11508–11515. 10.1021/bi980087p 9708986

[B29] Herlin M. Finnila M. A. Zioupos P. Aula A. Risteli J. Miettinen H. M. (2013). New insights to the role of aryl hydrocarbon receptor in bone phenotype and in dioxin-induced modulation of bone microarchitecture and material properties. *Toxicol. Appl. Pharmacol.* 273 219–226. 10.1016/j.taap.2013.09.002 24035824

[B30] Hou Q. Ye L. Liu H. Huang L. Yang Q. Turner J. R. (2018). Lactobacillus accelerates ISCs regeneration to protect the integrity of intestinal mucosa through activation of STAT3 signaling pathway induced by LPLs secretion of IL-22. *Cell Death Differ.* 25 1657–1670. 10.1038/s41418-018-0070-2 29459771 PMC6143595

[B31] Hsieh H. L. Yang C. M. (2013). Role of redox signaling in neuroinflammation and neurodegenerative diseases. *BioMed Res. Int.* 2013:484613. 10.1155/2013/484613 PMC3884773 24455696

[B32] Huang Y. Zhou J. Wang S. Xiong J. Chen Y. Liu Y. (2020). Indoxyl sulfate induces intestinal barrier injury through IRF1-DRP1 axis-mediated mitophagy impairment. *Theranostics* 10 7384–7400. 10.7150/thno.45455 32641998 PMC7330852

[B33] Hubbard T. D. Murray I. A. Perdew G. H. (2015). Indole and tryptophan metabolism: endogenous and dietary routes to Ah receptor activation. *Drug Metab. Dispos.* 43 1522–1535. 10.1124/dmd.115.064246 26041783 PMC4576673

[B34] Humblet O. Birnbaum L. Rimm E. Mittleman M. A. Hauser R. (2008). Dioxins and cardiovascular disease mortality. *Environ. Health Perspect.* 116 1443–1448. 10.1289/ehp.11579 19057694 PMC2592261

[B35] Ichii O. Otsuka-Kanazawa S. Nakamura T. Ueno M. Kon Y. Chen W. (2014). Podocyte injury caused by indoxyl sulfate, a uremic toxin and aryl-hydrocarbon receptor ligand. *PLoS One* 9:e108448. 10.1371/journal.pone.0108448 25244654 PMC4171541

[B36] Ikizler T. A. Cano N. J. Franch H. Fouque D. Himmelfarb J. Kalantar-Zadeh K. (2013). Prevention and treatment of protein energy wasting in chronic kidney disease patients: a consensus statement by the International Society of Renal Nutrition and Metabolism. *Kidney Int.* 84 1096–1107. 10.1038/ki.2013.147 23698226

[B37] Ito S. Osaka M. Edamatsu T. Itoh Y. Yoshida M. (2016). Crucial role of the Aryl hydrocarbon receptor (AhR) in indoxyl sulfate-induced vascular inflammation. *J. Atheroscler. Thromb.* 23 960–975. 10.5551/jat.34462 26860885 PMC7399298

[B38] Izawa T. Arakaki R. Mori H. Tsunematsu T. Kudo Y. Tanaka E. (2016). The nuclear receptor AhR controls bone homeostasis by regulating osteoclast differentiation via the RANK/c-Fos signaling axis. *J. Immunol.* 197 4639–4650. 10.4049/jimmunol.1600822 27849171 PMC5133671

[B39] Jeuken A. Keser B. J. Khan E. Brouwer A. Koeman J. Denison M. S. (2003). Activation of the Ah receptor by extracts of dietary herbal supplements, vegetables, and fruits. *J. Agric. Food Chem.* 51 5478–5487. 10.1021/jf030252u 12926901

[B40] Kalaska B. Pawlak K. Domaniewski T. Oksztulska-Kolanek E. Znorko B. Roszczenko A. (2017). Elevated levels of peripheral kynurenine decrease bone strength in rats with chronic kidney disease. *Front. Physiol.* 8:836. 10.3389/fphys.2017.00836 29163188 PMC5671515

[B41] Kennedy P. J. Cryan J. F. Dinan T. G. Clarke G. (2017). Kynurenine pathway metabolism and the microbiota-gut-brain axis. *Neuropharmacology* 112 399–412. 10.1016/j.neuropharm.2016.07.002 27392632

[B42] Kidney Disease: Improving Global Outcomes (KDIGO) CKD-MBD Update Work Group (2017). KDIGO 2017 clinical practice guideline update for the diagnosis, evaluation, prevention, and treatment of chronic kidney disease-mineral and bone disorder (CKD-MBD). *Kidney Int. Suppl.* 7 1–59. 10.1016/j.kisu.2017.04.001 30675420 PMC6340919

[B43] Kim J. T. Kim S. H. Min H. K. Jeon S. J. Sung S. A. Park W. H. (2020). Effect of dialysis on Aryl hydrocarbon receptor transactivating activity in patients with chronic kidney disease. *Yonsei Med. J.* 61 56–63. 31887800 10.3349/ymj.2020.61.1.56 PMC6938787

[B44] Kim J. T. Kim S. S. Jun D. W. Hwang Y. H. Park W. H. Pak Y. K. (2013). Serum arylhydrocarbon receptor transactivating activity is elevated in type 2 diabetic patients with diabetic nephropathy. *J. Diabetes Investig.* 4 483–491. 10.1111/jdi.12081 24843699 PMC4025111

[B45] Kim S. Y. Yang J. H. (2005). Neurotoxic effects of 2,3,7,8-tetrachlorodibenzo-p-dioxin in cerebellar granule cells. *Exp. Mol. Med.* 37 58–64. 10.1038/emm.2005.8 15761253

[B46] Kimura A. Naka T. Nohara K. Fujii-Kuriyama Y. Kishimoto T. (2008). Aryl hydrocarbon receptor regulates Stat1 activation and participates in the development of Th17 cells. *Proc. Natl. Acad. Sci. U.S.A.* 105 9721–9726. 10.1073/pnas.0804231105 18607004 PMC2474493

[B47] Koizumi M. Tatebe J. Watanabe I. Yamazaki J. Ikeda T. Morita T. (2014). Aryl hydrocarbon receptor mediates indoxyl sulfate-induced cellular senescence in human umbilical vein endothelial cells. *J. Atheroscler. Thromb.* 21 904–916. 10.5551/jat.23663 24727683

[B48] Lee W. J. Liu S. H. Chiang C. K. Lin S. Y. Liang K. W. Chen C. H. (2016). Aryl hydrocarbon receptor deficiency attenuates oxidative stress-related mesangial cell activation and macrophage infiltration and extracellular matrix accumulation in diabetic nephropathy. *Antioxid. Redox Signal.* 24 217–231. 10.1089/ars.2015.6310 26415004

[B49] Levin A. Djurdjev O. Duncan J. Rosenbaum D. Werb R. (2006). Haemoglobin at time of referral prior to dialysis predicts survival: an association of haemoglobin with long-term outcomes. *Nephrol. Dial. Transplant.* 21 370–377. 10.1093/ndt/gfi209 16249203

[B50] Li S. Bostick J. W. Ye J. Qiu J. Zhang B. Urban J. F. Jr. (2018). Aryl hydrocarbon receptor signaling cell intrinsically inhibits intestinal group 2 innate lymphoid cell function. *Immunity* 49 915–928.e5. 30446384 10.1016/j.immuni.2018.09.015 PMC6249058

[B51] Lin C. H. Juan S. H. Wang C. Y. Sun Y. Y. Chou C. M. Chang S. F. (2008). Neuronal activity enhances aryl hydrocarbon receptor-mediated gene expression and dioxin neurotoxicity in cortical neurons. *J. Neurochem.* 104 1415–1429. 10.1111/j.1471-4159.2007.05098.x 17973980

[B52] Lindsey S. Jiang J. Woulfe D. Papoutsakis E. T. (2014). Platelets from mice lacking the aryl hydrocarbon receptor exhibit defective collagen-dependent signaling. *J. Thromb. Haemost.* 12 383–394. 10.1111/jth.12490 24410994 PMC4008149

[B53] Lindsey S. Papoutsakis E. T. (2011). The aryl hydrocarbon receptor (AHR) transcription factor regulates megakaryocytic polyploidization. *Br. J. Haematol.* 152 469–484. 10.1111/j.1365-2141.2010.08548.x 21226706 PMC3408620

[B54] Lipnicki D. M. Crawford J. Kochan N. A. Trollor J. N. Draper B. Reppermund S. (2017). Risk factors for mild cognitive impairment, dementia and mortality: the sydney memory and ageing study. *J. Am. Med. Dir. Assoc.* 18 388–395. 28043804 10.1016/j.jamda.2016.10.014

[B55] Lowenstein J. (2014). Agent Orange and heart disease: Is there a connection? *FASEB J.* 28 1531–1533. 10.1096/fj.14-0402ufm 24688078

[B56] Lu H. Lei X. Klaassen C. (2006). Gender differences in renal nuclear receptors and aryl hydrocarbon receptor in 5/6 nephrectomized rats. *Kidney Int.* 70 1920–1928. 10.1038/sj.ki.5001880 16985511

[B57] Mafra D. Borges N. A. Lindholm B. Shiels P. G. Evenepoel P. Stenvinkel P. (2020). Food as medicine: targeting the uraemic phenotype in chronic kidney disease. *Nat. Rev. Nephrol.* 10.1038/s41581-020-00345-8 [Epub ahead of print]. 32963366

[B58] Manfredo Vieira S. Hiltensperger M. Kumar V. Zegarra-Ruiz D. Dehner C. Khan N. (2018). Translocation of a gut pathobiont drives autoimmunity in mice and humans. *Science* 359 1156–1161. 10.1126/science.aar7201 29590047 PMC5959731

[B59] Mascanfroni I. D. Takenaka M. C. Yeste A. Patel B. Wu Y. Kenison J. E. (2015). Metabolic control of type 1 regulatory T cell differentiation by AHR and HIF1-alpha. *Nat. Med.* 21 638–646. 10.1038/nm.3868 26005855 PMC4476246

[B60] McIntosh B. E. Hogenesch J. B. Bradfield C. A. (2010). Mammalian Per-Arnt-Sim proteins in environmental adaptation. *Annu. Rev. Physiol.* 72 625–645. 10.1146/annurev-physiol-021909-135922 20148691

[B61] Meijers B. Evenepoel P. Anders H. J. (2019). Intestinal microbiome and fitness in kidney disease. *Nat. Rev. Nephrol.* 15 531–545. 10.1038/s41581-019-0172-1 31243394

[B62] Meijers B. K. Evenepoel P. (2011). The gut-kidney axis: indoxyl sulfate, p-cresyl sulfate and CKD progression. *Nephrol. Dial. Transplant.* 26 759–761. 10.1093/ndt/gfq818 21343587

[B63] Metidji A. Omenetti S. Crotta S. Li Y. Nye E. Ross E. (2018). The environmental sensor AHR protects from inflammatory damage by maintaining intestinal stem cell homeostasis and barrier integrity. *Immunity* 49 353–362.e5. 30119997 10.1016/j.immuni.2018.07.010 PMC6104739

[B64] Mohammadi S. Seyedhosseini F. S. Behnampour N. Yazdani Y. (2017). Indole-3-carbinol induces G1 cell cycle arrest and apoptosis through aryl hydrocarbon receptor in THP-1 monocytic cell line. *J. Recept. Signal Transduct. Res.* 37 506–514. 10.1080/10799893.2017.1360351 28812970

[B65] Mohanram A. Zhang Z. Shahinfar S. Keane W. F. Brenner B. M. Toto R. D. (2004). Anemia and end-stage renal disease in patients with type 2 diabetes and nephropathy. *Kidney Int.* 66 1131–1138. 10.1111/j.1523-1755.2004.00863.x 15327408

[B66] Murray I. A. Patterson A. D. Perdew G. H. (2014). Aryl hydrocarbon receptor ligands in cancer: friend and foe. *Nat. Rev. Cancer* 14 801–814. 10.1038/nrc3846 25568920 PMC4401080

[B67] Mutsaers H. A. Caetano-Pinto P. Seegers A. E. Dankers A. C. van den Broek P. H. Wetzels J. F. (2015a). Proximal tubular efflux transporters involved in renal excretion of p-cresyl sulfate and p-cresyl glucuronide: implications for chronic kidney disease pathophysiology. *Toxicol. In Vitro* 29 1868–1877. 10.1016/j.tiv.2015.07.020 26216510

[B68] Mutsaers H. A. Stribos E. G. Glorieux G. Vanholder R. Olinga P. (2015b). Chronic kidney disease and fibrosis: the role of uremic retention solutes. *Front. Med.* 2:60. 10.3389/fmed.2015.00060 26380262 PMC4553389

[B69] Nemeth E. Tuttle M. S. Powelson J. Vaughn M. B. Donovan A. Ward D. M. (2004). Hepcidin regulates cellular iron efflux by binding to ferroportin and inducing its internalization. *Science* 306 2090–2093. 10.1126/science.1104742 15514116

[B70] Ng H. Y. Yisireyili M. Saito S. Lee C. T. Adelibieke Y. Nishijima F. (2014). Indoxyl sulfate downregulates expression of Mas receptor via OAT3/AhR/Stat3 pathway in proximal tubular cells. *PLoS One* 9:e91517. 10.1371/journal.pone.0091517 24614509 PMC3948887

[B71] Nihei T. Miura Y. Yagasaki K. (2001). Inhibitory effect of resveratrol on proteinuria, hypoalbuminemia and hyperlipidemia in nephritic rats. *Life Sci.* 68 2845–2852. 10.1016/s0024-3205(01)01061-x 11432450

[B72] Obata Y. Castano A. Boeing S. Bon-Frauches A. C. Fung C. Fallesen T. (2020). Neuronal programming by microbiota regulates intestinal physiology. *Nature* 578 284–289. 10.1038/s41586-020-1975-8 32025031

[B73] Ochi A. Mori K. Nakatani S. Emoto M. Morioka T. Motoyama K. (2015). Indoxyl sulfate suppresses hepatic fetuin-A expression via the aryl hydrocarbon receptor in HepG2 cells. *Nephrol. Dial. Transplant.* 30 1683–1692. 10.1093/ndt/gfv250 26068716

[B74] Opitz C. A. Litzenburger U. M. Sahm F. Ott M. Tritschler I. Trump S. (2011). An endogenous tumour-promoting ligand of the human aryl hydrocarbon receptor. *Nature* 478 197–203. 10.1038/nature10491 21976023

[B75] Park C. H. Valore E. V. Waring A. J. Ganz T. (2001). Hepcidin, a urinary antimicrobial peptide synthesized in the liver. *J. Biol. Chem.* 276 7806–7810. 10.1074/jbc.m008922200 11113131

[B76] Piccinni M. P. Lombardelli L. Logiodice F. Kullolli O. Maggi E. Barkley M. S. (2019). Medroxyprogesterone acetate decreases Th1, Th17, and increases Th22 responses via AHR signaling which could affect susceptibility to infections and inflammatory disease. *Front. Immunol.* 10 642. 10.3389/fimmu.2019.00642 31001262 PMC6456711

[B77] Pollenz R. S. (2002). The mechanism of AH receptor protein down-regulation (degradation) and its impact on AH receptor-mediated gene regulation. *Chem. Biol. Interact.* 141 41–61. 10.1016/s0009-2797(02)00065-0 12213384

[B78] Pombo M. Lame M. W. Walker N. J. Huynh D. H. Tablin F. (2015). TCDD and omeprazole prime platelets through the aryl hydrocarbon receptor (AhR) non-genomic pathway. *Toxicol. Lett.* 235 28–36. 10.1016/j.toxlet.2015.03.005 25797602

[B79] Pongratz I. Mason G. G. Poellinger L. (1992). Dual roles of the 90-kDa heat shock protein hsp90 in modulating functional activities of the dioxin receptor. Evidence that the dioxin receptor functionally belongs to a subclass of nuclear receptors which require hsp90 both for ligand binding activity and repression of intrinsic DNA binding activity. *J. Biol. Chem.* 267 13728–13734. 1320028

[B80] Puga A. Ma C. Marlowe J. L. (2009). The aryl hydrocarbon receptor cross-talks with multiple signal transduction pathways. *Biochem. Pharmacol.* 77 713–722. 10.1016/j.bcp.2008.08.031 18817753 PMC2657192

[B81] Quintana F. J. Yeste A. Mascanfroni I. D. (2015). Role and therapeutic value of dendritic cells in central nervous system autoimmunity. *Cell Death Differ.* 22 215–224. 10.1038/cdd.2014.125 25168240 PMC4291485

[B82] Ramezani A. Raj D. S. (2014). The gut microbiome, kidney disease, and targeted interventions. *J. Am. Soc. Nephrol.* 25 657–670. 10.1681/asn.2013080905 24231662 PMC3968507

[B83] Refaey M. E. McGee-Lawrence M. E. Fulzele S. Kennedy E. J. Bollag W. B. Elsalanty M. (2017). Kynurenine, a tryptophan metabolite that accumulates with age, induces bone loss. *J. Bone Miner. Res.* 32 2182–2193. 10.1002/jbmr.3224 28727234 PMC5685888

[B84] Rothhammer V. Quintana F. J. (2019). The aryl hydrocarbon receptor: an environmental sensor integrating immune responses in health and disease. *Nat. Rev. Immunol.* 19 184–197. 10.1038/s41577-019-0125-8 30718831

[B85] Sallee M. Dou L. Cerini C. Poitevin S. Brunet P. Burtey S. (2014). The aryl hydrocarbon receptor-activating effect of uremic toxins from tryptophan metabolism: a new concept to understand cardiovascular complications of chronic kidney disease. *Toxins* 6 934–949. 10.3390/toxins6030934 24599232 PMC3968369

[B86] Santana M. T. Poitevin S. Paul P. McKay N. Jourde-Chiche N. Legris T. (2018). Indoxyl sulfate upregulates liver P-glycoprotein expression and activity through aryl hydrocarbon receptor signaling. *J. Am. Soc. Nephrol.* 29 906–918. 29222397 10.1681/ASN.2017030361 PMC5827590

[B87] Schroeder J. C. Dinatale B. C. Murray I. A. Flaveny C. A. Liu Q. Laurenzana E. M. (2010). The uremic toxin 3-indoxyl sulfate is a potent endogenous agonist for the human aryl hydrocarbon receptor. *Biochemistry* 49 393–400. 10.1021/bi901786x 20000589 PMC2805781

[B88] Shivanna S. Kolandaivelu K. Shashar M. Belghasim M. Al-Rabadi L. Balcells M. (2016). The Aryl hydrocarbon receptor is a critical regulator of tissue factor stability and an antithrombotic target in uremia. *J. Am. Soc. Nephrol.* 27 189–201. 10.1681/asn.2014121241 26019318 PMC4696580

[B89] Singh R. Chandrashekharappa S. Bodduluri S. R. Baby B. V. Hegde B. Kotla N. G. (2019). Enhancement of the gut barrier integrity by a microbial metabolite through the Nrf2 pathway. *Nat. Commun.* 10:89. 10.1038/s41467-018-07859-7 PMC6327034 30626868

[B90] Sirich T. L. (2017). Obstacles to reducing plasma levels of uremic solutes by hemodialysis. *Semin. Dial.* 30 403–408. 10.1111/sdi.12609 28558415

[B91] Sonnenberg G. F. Fouser L. A. Artis D. (2011). Border patrol: regulation of immunity, inflammation and tissue homeostasis at barrier surfaces by IL-22. *Nat. Immunol.* 12 383–390. 10.1038/ni.2025 21502992

[B92] Soshilov A. Denison M. S. (2008). Role of the Per/Arnt/Sim domains in ligand-dependent transformation of the aryl hydrocarbon receptor. *J. Biol. Chem.* 283 32995–33005. 10.1074/jbc.m802414200 18806268 PMC2583286

[B93] Souma T. Nezu M. Nakano D. Yamazaki S. Hirano I. Sekine H. (2016). Erythropoietin synthesis in renal myofibroblasts is restored by activation of hypoxia signaling. *J. Am. Soc. Nephrol.* 27 428–438. 10.1681/asn.2014121184 26054543 PMC4731118

[B94] Souma T. Suzuki N. Yamamoto M. (2015). Renal erythropoietin-producing cells in health and disease. *Front. Physiol.* 6:167. 10.3389/fphys.2015.00167 26089800 PMC4452800

[B95] Souma T. Yamazaki S. Moriguchi T. Suzuki N. Hirano I. Pan X. (2013). Plasticity of renal erythropoietin-producing cells governs fibrosis. *J. Am. Soc. Nephrol.* 24 1599–1616. 10.1681/asn.2013010030 23833259 PMC3785278

[B96] Strassel C. Brouard N. Mallo L. Receveur N. Mangin P. Eckly A. (2016). Aryl hydrocarbon receptor-dependent enrichment of a megakaryocytic precursor with a high potential to produce proplatelets. *Blood* 127 2231–2240. 10.1182/blood-2015-09-670208 26966088 PMC4859197

[B97] Tampe B. Tampe D. Nyamsuren G. Klopper F. Rapp G. Kauffels A. (2018). Pharmacological induction of hypoxia-inducible transcription factor ARNT attenuates chronic kidney failure. *J. Clin. Investig.* 128 3053–3070. 10.1172/jci89632 29664738 PMC6025987

[B98] Vanholder R. Fouque D. Glorieux G. Heine G. H. Kanbay M. Mallamaci F. (2016). Clinical management of the uraemic syndrome in chronic kidney disease. *Lancet Diabetes Endocrinol.* 4 360–373. 26948372 10.1016/S2213-8587(16)00033-4

[B99] Vanholder R. Glorieux G. (2014). Introduction: uremic toxicity - state of the Art 2014. *Semin. Nephrol.* 34 85–86. 10.1016/j.semnephrol.2014.02.001 26248716

[B100] Vidal C. Li W. Santner-Nanan B. Lim C. K. Guillemin G. J. Ball H. J. (2015). The kynurenine pathway of tryptophan degradation is activated during osteoblastogenesis. *Stem Cells* 33 111–121. 10.1002/stem.1836 25186311

[B101] Wakamatsu T. Yamamoto S. Ito T. Sato Y. Matsuo K. Takahashi Y. (2018). Indoxyl sulfate promotes macrophage IL-1beta production by activating Aryl hydrocarbon receptor/NF-kappa/MAPK cascades, but the NLRP3 inflammasome Was Not Activated. *Toxins* 10:124. 10.3390/toxins10030124 29543732 PMC5869412

[B102] Walker J. A. Richards S. Belghasem M. E. Arinze N. Yoo S. B. Tashjian J. Y. (2020). Temporal and tissue-specific activation of aryl hydrocarbon receptor in discrete mouse models of kidney disease. *Kidney Int.* 97 538–550. 10.1016/j.kint.2019.09.029 31932072 PMC9721455

[B103] Wang G. Z. Zhang L. Zhao X. C. Gao S. H. Qu L. W. Yu H. (2019). The Aryl hydrocarbon receptor mediates tobacco-induced PD-L1 expression and is associated with response to immunotherapy. *Nat. Commun.* 10:1125. 10.1038/s41467-019-08887-7 PMC6408580 30850589

[B104] Wang X. Ota N. Manzanillo P. Kates L. Zavala-Solorio J. Eidenschenk C. (2014). Interleukin-22 alleviates metabolic disorders and restores mucosal immunity in diabetes. *Nature* 514 237–241. 10.1038/nature13564 25119041

[B105] Watanabe I. Tatebe J. Namba S. Koizumi M. Yamazaki J. Morita T. (2013). Activation of aryl hydrocarbon receptor mediates indoxyl sulfate-induced monocyte chemoattractant protein-1 expression in human umbilical vein endothelial cells. *Circ. J.* 77 224–230. 10.1253/circj.cj-12-0647 23037589

[B106] Williamson M. A. Gasiewicz T. A. Opanashuk L. A. (2005). Aryl hydrocarbon receptor expression and activity in cerebellar granule neuroblasts: implications for development and dioxin neurotoxicity. *Toxicol. Sci.* 83 340–348. 10.1093/toxsci/kfi031 15537747

[B107] Xia J. Lin J. Li X. N. Zhang C. Li N. Du Z. H. (2018). Atrazine-induced environmental nephrosis was mitigated by lycopene via modulating nuclear xenobiotic receptors-mediated response. *J. Nutr. Biochem.* 51 80–90. 10.1016/j.jnutbio.2017.09.006 29107825

[B108] Yano J. M. Yu K. Donaldson G. P. Shastri G. G. Ann P. Ma L. (2015). Indigenous bacteria from the gut microbiota regulate host serotonin biosynthesis. *Cell* 161 264–276. 10.1016/j.cell.2015.02.047 25860609 PMC4393509

[B109] Yu M. Wang Q. Ma Y. Li L. Yu K. Zhang Z. (2018). Aryl hydrocarbon receptor activation modulates intestinal epithelial barrier function by maintaining tight junction integrity. *Int. J. Biol. Sci.* 14 69–77. 10.7150/ijbs.22259 29483826 PMC5821050

[B110] Zaher H. Fernandez-Salguero P. M. Letterio J. Sheikh M. S. Fornace A. J. Jr. Roberts A. B. (1998). The involvement of aryl hydrocarbon receptor in the activation of transforming growth factor-beta and apoptosis. *Mol. Pharmacol.* 54 313–321. 10.1124/mol.54.2.313 9687573

[B111] Zelante T. Iannitti R. G. Cunha C. De Luca A. Giovannini G. Pieraccini G. (2013). Tryptophan catabolites from microbiota engage aryl hydrocarbon receptor and balance mucosal reactivity via interleukin-22. *Immunity* 39 372–385. 10.1016/j.immuni.2013.08.003 23973224

[B112] Zhao B. Bohonowych J. E. Timme-Laragy A. Jung D. Affatato A. A. Rice R. H. (2013). Common commercial and consumer products contain activators of the aryl hydrocarbon (dioxin) receptor. *PLoS One* 8:e56860. 10.1371/journal.pone.0056860 23441220 PMC3575475

